# Two cases of pre-extensively drug resistant tuberculosis in children in Indonesia

**DOI:** 10.1016/j.rmcr.2021.101544

**Published:** 2021-11-12

**Authors:** Heda Melinda Nataprawira, Indah Septiane, Sri Sudarwati, Diah Asri Wulandari

**Affiliations:** Department of Child Health, Hasan Sadikin General Hospital/Faculty of Medicine, Universitas Padjadjaran, Indonesia

**Keywords:** Pre-extensively drug resistant tuberculosis, Children, Case report

## Abstract

Few reports are available on children with pre-extensively drug-resistant tuberculosis (pre-XDR-TB), which is defined as *Mycobacterium tuberculosis* resistant to both isoniazid and rifampicin plus resistance to either a fluoroquinolone or a second-line injectable drug. Pre-XDR-TB treatment for children usually has been individualized based on drug susceptibility test (DST) results, but treatment remains challenging due to the lack of studies based on existing treatment guidelines in children and lack of availability of the new drugs. We report two cases of pre-XDR-TB in children who have responded well to individualized treatment regimens. Because toxic drugs are used for long duration, close monitoring of adverse drug reactions is important.

## 1 Introduction

The incidence of drug–resistant tuberculosis is increasing in many areas of the world [1]. Drug resistant tuberculosis is devided in to several types such as monoresistant, polydrug-resistant, multidrug-resistant (MDR), pre-extensively drug resistant (pre-XDR), extensively drug resistant (XDR) [1,2]. In 2016, a total of 8014 cases of XDR-TB were reported to the WHO by 72 countries [3]. Estimates suggest that 650.000 children with MDR-TB, 4.7% have XDR TB, unfortunately there is still lack data of pre–XDR cases reported in children [4]. Pre-XDR TB is MDR TB plus resistance to fluoroquinolone or second line injectable drugs (capreomycin, amikacin, kanamycin) [5]. Pre-XDR TB is confirmed by *M.tuberculosis* culture and drug susceptibility test (DST) either genotypically (by first- and second-line line-probe assays or whole-genome sequencing) or phenotypically (by culture-based DST) [5,6]. The management of pre-XDR TB in children remains challenging because of the limited availability of the new drugs and appropriate treatment regimens guidelines. Pre–XDR TB treatment regimen for children have historically been individualized on the basis of LPA or mycobacterial DST of the organism of the child. The long duration treatment of Pre-XDR TB has lead to several adverse reactions which should be monitored frequently [7,8]. We present two cases of children diagnosed as pre-XDR TB who received individualized strategy treatment based on their culture and DST result.

## 2 Case 1

**Fig. 1:**
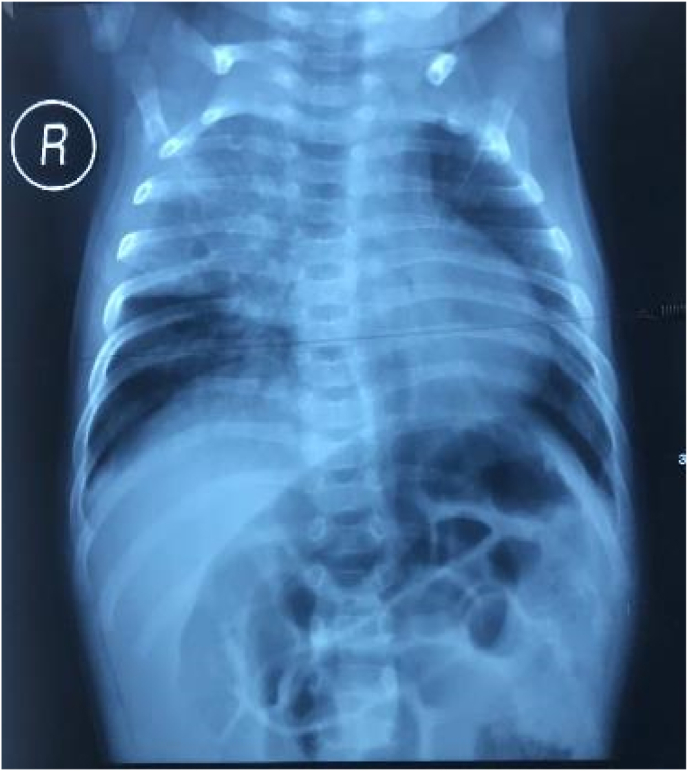
Chest X-Ray of a 3-months-old boy with Pre-XDR TB. It shows infiltrate at the upper-middle right lung field with an increased of bronchovascular marking.

A–3 months-old-boy presented with severe malnutrition, known cholestatic jaundice diagnosed as congenital cytomegalovirus infection and contact of his father who was diagnosed with MDR-TB but was not compliant with his treatment. His tuberculin skin test was positive, chest radiograph revealed right perihilar infiltrate and X-pert MTB/RIF on gastric aspirate was positive for *M. tuberculosis* complex with resistance to rifampicin. Other baseline investigations revealed a small atrial septal defect on echocardiography, mild to moderate hearing loss on audiology, normal vision and retinas on ophthalmological evaluation, raised bilirubin and liver enzymes (alanine and aspartate transaminases) and positive cytomegalovirus (CMV) IgM with a negative CMV PCR. He was HIV-unexposed and his HIV ELISA was negative, but CD4% was low (19%). Culture of the gastric aspirate eventually was positive, confirming *M. tuberculosis* resistant to isoniazid and rifampicin on line-probe assay as well as resistant to second-line injectable agents with second-line LPA, but susceptible to the fluoroquinolones. He was therefore confirmed as pre-XDR-TB. The mother's sputum was X-pert MTB/RIF negative. The chest x-ray showed active tuberculosis with infiltrates at the right upper-middle lung field with an increased of bronchovascular marking (Fig. 1). We treated him with five kind of less hepatotoxic anti tuberculosis drugs by individualized treatment based on the updated WHO 2018 guidelines. He commenced the treatment with moxifloxacin (10 mg/kg/day divided in tow dose), linezolid (10 mg/kg/day once daily), cycloserin (10 mg/kg/day once daily), ethambutol (20 mg/kg/day once daily) and para-aminosalysilic acid (200 mg/kg/day divided in two dose). After seven days receiving the pre- XDR-TB treatment, the liver enzymes improved, no adverse reaction such as vomiting, dhiarrea was reported. He also recieved gancyclovir for the CMV infection. He was followed every one month, he shows weight increment to 5.8 kg from 3.6 kg in 3 months and increased gradually every month. His nutritional status improved to normal from severe malnutrition after 20 months of treatment. No adverse reaction such as vomiting nor dhiarrea was reported. The growth and milestones development were appropriate. Laboratory evaluation showed improvement of liver function. Hematologic and neurologic (peripheral and toxic optic neuropathy) adverse effects were monitored during linezolid treatment. No anemia nor thrombocytopenia was found. For the optic toxic neuropathy evaluation, normal vision was concluded through examination of response to light, pupil response, ability to follow a target and ophthalmoscopy examination of the retina was also done to exclude CMV retinitis symptom and the result was normal. The *M.tuberculosis* culture evaluation result was negative on the first and third month of treatment.

## 3 Case 2

**Fig. 2:**
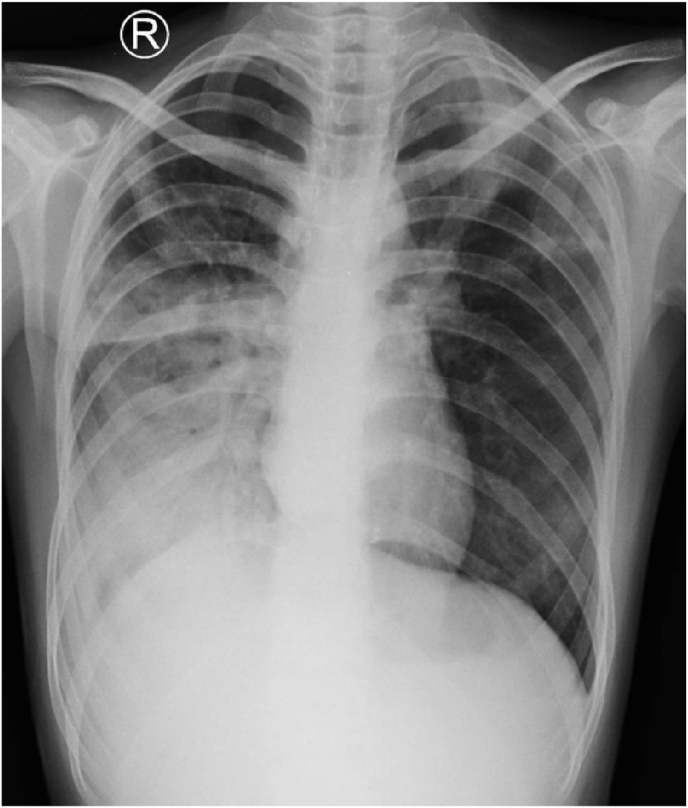
Chest X-Ray of a 14-years-olf girl with Pre-XDR TB. It shows opacity at the right hilar, lobulated infiltrate in the left apex, nodular at left hilar, and lymph node enlargement at bilateral peri-hilar.

A–14 years-old-girl presented with severe weight loss (severe malnutrition), with chief complaint of fever since 1 month and hemoptysis since 2 days prior. Her parents showed negative for TB screening. History of tuberculosis contact is her neighbour identified MDR-TB and received treatment in our hospital. Her X-pert MTB/RIF revealed positive *Mycobacterium tuberculosis* with Rifampicin resistant. Culture *M. tuberculosis* was positive, confirming *M. tuberculosis* resistant to isoniazid and rifampicin on line-probe assay as well as resistant to second-line injectable agents with second-line LPA, but susceptible to the fluoroquinolones. The chest x-ray showed active tuberculosis with opacity at the right hilar, lobulated infiltrate in the left apex, nodular at left hilar, and enlarged bilateral peri-hilar lymph nodes (Fig. 2). Before starting the therapy she was consulted to the Psychiatry, Ophthalmology and Ear, Nose and Throat department. Baseline electrocardiography (ECG) showed normal QT interval. HIV screening was negative. She started on individualized drugs regimen for pre–XDR TB with levofloxacin (10 mg/kg/day once daily), ethionamide (15 mg/kg/day once daily), cycloserine (10 mg/kg/day once daily), pyrazinamide (35 mg/kg/day once daily), para-aminosalicylic acid (PAS 200 mg/kg/day divided in two dose), bedaquiline (200 mg once daily for 2 weeks). On the second day receiving bedaquiline, the ECG showed prolonged QT interval >500 ms without any electrolyte imbalance, so bedaquiline was stopped and ECG was examined every day. She started to receive linezolid 400 mg per day (10 mg/kg/day once daily) replacing bedaquiline. There was no more prolonged QT after given linezolid. Laboratory examination and clinical manifestation was monitored due to the side effects of the therapy. During hospitalization no other adverse reaction occurred. The laboratory examination is within normal limit. After two weeks hospitalized, she was discharged. She was followed every one month, no adverse reaction of nausea, vomit, jaundice was reported. Linezolid toxicity was also observed during treatment, hematologic value was normal, no anemia nor thrombocytopenia was found, there were no vision loss and color vision test result was normal (evaluated through Ishihara test). No peripheral neuropathy signs (paresthesia, numbness in extremities) were reported. Her weight increased 2 kg after 3 months treatment and her nutritional status improved to normal weight from severe malnutrition after 20 months of treatment. The *M.tuberculosis* culture result was negative on the first and third month of treatment.

## 4 Discussion

We reported two cases of children diagnosed as pre–XDR TB. To our knowledge, this is the first cases of pre–XDR TB in children in Indonesia. The incidence of drug–resistant tuberculosis is increasing in many areas of the world [1]. There are two major in types of drug resistance. Pre-XDR TB is confirmed by *M.tuberculosis* culture, line probe assays (LPA) and drug susceptibility test (DST). The LPA is a family of DNA strip-based test that determine of amplicons (DNA amplification products) to probes targeting the most common resistance associated mutations to first and second line drugs. Line probe assay demonstrated high sensitivity 96.7% and high specificity 98.8% for detecting pre-XDR TB and XDR-TB [6,7]. Phenotypic drug sensitivity testing (DST) in these case uses the liquid culture BACTEC *Mycobacterium* growth indicator tubes (MGIT) 960 system. The BACTEC MGIT 960 system is fully automated, detects high volume mycobacteria growth, detection and susceptibility testing. The results revealed in 9–15 days [8]. On both cases, the DST result was resistance to TB second line injectable drugs (kanamycin, capreomycin, amikacin) but susceptible to fluoroquinolones. Thus, in our case, both of them were diagnosed and treated based on the LPA and DST result, and the *M. tuberculosis* culture result was positive. Clinically, MDR-TB must be considered in children with a clinical manifestation of tuberculosis with several condition: history of tuberculosis 6–12 months prior, no improvement after first line medication for 2–3 months, contact with TB patient who died during treatment, and treatment failed [9]. In our case, both of them were suspected MDR-TB due to the history of MDR-TB contact. The management of pre-XDR TB in children remains challenging because of the limited availability of the new drugs and appropriate treatment regimens guidelines [9,10]. Pre–XDR TB treatment regimen for children have historically been individualized on the basis of LPA or mycobacterial DST of the organism of the child. WHO describes treatment strategies for MDR and pre–XDR TB is divided into standardized treatment, drug resistance surveillance (DRS) data from representative patient population are used to as the basis for regimen design in the absence of individual DST, standardized treatment is classified into conventional (20–26 months) and short term treatment (9–11 months). Individualized treatment is each regimen designed based on the patient's past history of TB treatment, and individual DST result, this strategy is used for the pre-XDR and XDR treatment [3,11,12]. In our case the patient received regimen that is based on the LPA and DSTresult. Individualized treatment should be given for at least 18–24 months of duration [10,11]. There are lack data on the optimal combination of medications and duration of treatment for pre-XDR TB in children. A systematic review reported treatment outcomes in 37 children with XDR-TB, 81% of them had a successful treatment outcome (cured and complete treatment), as defined by WHO guidelines [4]. Even though we still can't defined the treatment outcome in our patient, treatment outcomes were defined by using standard 2016 WHO MDR TB outcome definitions as classified by treating clinicians: cured (treatment completed as recommended by the national policy without evidence of failure and >3 consecutive cultures taken at least 30 days apart were negative after the intensive phase of treatment); treatment complete (as for cure but without records of negative cultures); treatment failed (treatment stopped or requiring change of 2 drugs because of persistent positive cultures at end of the intensive phase or reversion to positive cultures in the continuation phase, or evidence of additional acquired resistance or adverse drug reactions); death (for any reason while receiving treatment); or loss to follow up (treatment interruption for 2 consecutive months) [3,11]. Treatment was given based on the updated WHO 2018 guidelines, that regimen should be given at least five types of antituberculosis drug in intensive phase, four of second line that is proved still sensitive or never been used before, and one from the first line of antituberculosis drugs [1,2,12]. Every anti tuberculosis drugs was reported to have variable adverese reactions, one of them is hepatotoxic [13]. A systemic review and meta-analysis identified eight studies for a total of 315 patients with MDR-TB and pre-XDR TB, estimates for treatment success was 81.67% (95% CI), with the most common drug-related adverese events were nausea and vomiting. Other serious adverse events were hearing loss, psychiatric effects, and hypothyroidism. This suggests pre-XDR TB can be succesfully treated in children, with the overall proportion of children achieving treatment success [14]. In our first case, he had a previous elevated liver function due to cytomegalovirus infection so it was difficult to decide the best regimen for him. We started individualized regimen that is reported less hepatotoxic. The limitation of the first case is the negative result of the PCR-CMV, but this result might be influenced due to the examination that was taken after receiving 2 weeks of Gancyclovir due to the financial problems of his parents to attempt earlier examination. Mortality and defaulting seemed to be lower for children than for adults [14]. In our second case, adverse events that developed was prologed QT interval on the ECG due to bedaquiline, but it was improving after we altered to linezolid. Linezolid was reported useful and safe in treating children with pre-XDR TB [15,16]. No other adverse reactions occurred. Both of them are planned to have 18–24 months of treatment duration accordance to the WHO guidelines. Second line antituberculosis drugs used in pre-XDR TB have more adverse effects than the first line drugs. This, combined with large number of medications used for long duration, lead to frequently observed adverse effects in children on pre-XDR-TB treatment [17]. Although in this coronavirus (COVID-19) pandemic situation, both of them are assured to receive the treatment with good compliance based on the WHO guidelines to ensure continuity of essential services for all TB patients during the COVID-19 pandemic. Adequate availability TB medicines are provided to take home to ensure treatment completion without visiting hospitals or other treatment centres [18]. The limitation of our cases report is as the end of therapy outcomes were not assessed at the time of reporting this case, however interim outcomes were analyzed in details.

## 5 Conclusion

Treatment of pre-XDR TB for children remains challenging due to the lack data of treatment guidelines for children. Therefore it is important to treat children with pre–XDR TB by individualized strategy based on the clinical manifestations, line probe assays, and drug susceptibility testing result. Due to the long duration of treatment, close monitoring of adverse reaction should be done routinely. This pandemic COVID-19 situation should not be a barrier for the TB patients to receive a continuity treatment and monitoring.

## Declaration of competing interest

Non to declare.
